# Reproductive Toxicity Evaluation of Pestban Insecticide Exposure in Male and Female Rats

**DOI:** 10.5487/TR.2008.24.2.137

**Published:** 2008-06-01

**Authors:** Ashraf M. Morgan, A. M. Abd El-Aty

**Affiliations:** 17 Department of Toxicology and Forensic Medicine; Faculty of Veterinary Medicine, Cairo University, 12211 Giza, Egypt; 27 Department of Veterinary Pharmacology and Toxicology, College of Veterinary Medicine, Konkuk University, 1 Hwayang-dong, Kwangjin-gu, Seoul, 143-701 Korea; 37 Department of Pharmacology, Faculty of Veterinary Medicine, Cairo University, 12211 Giza, Egypt

**Keywords:** Pestban, Chlorpyrifos, Fertility, Reproduction, Rats

## Abstract

Sexually mature male and female rats were orally intubated with the organophosphorus insecticide, Pestban at a daily dosage of 7.45 or 3.72 mg/kg bwt, equivalent to 1/20 and 1/40 LD50, respectively. Male rats were exposed for 70 days, while the female rats were exposed for 14 days, premating, during mating and throughout the whole length of gestation and lactation periods till weaning. The results showed depressed acetylcholinesterase (AChE) activity in the brain of parents, fetuses and their placentae in a dose-dependent manner. The fertility was significantly reduced with increasing the dose in both treated groups, with more pronounced suppressive effects in the male treated group. The number of implantation sites and viable fetuses were significantly reduced in pregnant females of both treated groups. However, the number of resorptions, dead fetuses, and pre-and postimplantation losses were significantly increased. The incidence of resorptions was more pronounced in treated female compared to male group and was dose dependant. The behavioral responses as well as fetal survival and viability indices were altered in both treated groups during the lactation period. The incidence of these effects was more pronounced in the treated female group and occurred in a dose-related manner. The recorded morphological, visceral, and skeletal anomalies were significantly increased with increasing the dose in fetuses of both treated groups, with more pronounced effects on fetuses of treated females. In conclusion, the exposure of adult male and female rats to Pestban would cause adverse effects on fertility and reproduction.

## INTRODUCTION

Pesticides occupy a unique position among the many hazardous chemicals that man and animals encounter daily. Nowadays, insecticides especially organophosphorus insecticides (OPI) are extensively used in agriculture and in residential settings for combating insects (Whyatt *et al.*, 2002). They are powerful inhibitors of carboxylic ester hydrolases, including acetylcholinesterase (AChE) and butyrylcholinesterase (Karalliedde *et al.*, 2001). Moreover, prolonged exposure to these contaminants causes chronic neurologic syndrome, teratogenicity and male reproductive failure (Nafstad *et al.*, 1983). Some OPI are known to impair fertility, cause testicular degeneration, deteriorate semen quality in rodents (Bansal *et al.*, 1994) and bulls (Hass *et al.*, 1983), exert estrogenic (Garey and Wolff, 1998; Andersen *et al.*, 2002) and antiandrogenic (Tomura *et al.*, 2001; Kitamura *et al.*, 2003; Kojima *et al.*, 2004) activities.

Exposure of rodent dams during pregnancy to certain OPI, such as diazinon (Lozano *et al.*, 1989), phosphamidon (Soni and Bhatngar, 1989), dimethoate (Weber, 1990), chlorpyrifos (Chanda *et al.*, 1995), quinalphos (Srivastava *et al.*, 1992) and dimethoate (Srivastava and Raizada, 1996), has been associated with decrements in fetal growth, embryo/fetotoxicity and teratogenicity in some studies. Other studies on the same pesticides (Institoris *et al.*, 1995) and other organophosphates (Clemens *et al.*, 1990; Institoris *et al.*, 1995) have shown no association with fetal growth. Scanty animal studies have examined the relationship of length of gestation and organophosphate pesticide exposure. The few studies that have examined the association of prenatal pesticide exposure and fetal growth or gestational duration in humans have also shown conflicting results (Willis *et al.*, 1993; Kristensen *et al.*, 1997; Dabrowski *et al.*, 2003).

Pestban, the tested insecticide in our study is a product name for an organophosphorus insecticide containing 48% chlorpyrifos (CPF), an active ingredient widely used in agricultural practice. It is a cholinesterase inhibitor used against plants and household pests as well as to control cattle and sheep ectoparasites (Worthing and Walker, 1994). There is a growing body of literature suggesting a role for both acetylcholinesterase and butyrylcholinesterase in development (Layer and Willbold, 1995; Small *et al.*, 1996). Although CPF is a suspected developmental neurotoxicant (Dam *et al.*, 1998; Crumpton *et al.*, 2000; Raines *et al.*, 2001) and exerts some of its effects through cholinesterase inhibition, recent studies showed transplacental disposition and teratogenic effects of CPF when female rats were exposed to high CPF levels (Farag *et al.*, 2003; Akhtar *et al.*, 2006). Moreover, CPF is known to cross the placental barrier and inhibit AChE in the fetuses especially in the fetal brain (Dam *et al.*, 1998; Akhtar *et al.*, 2006). These toxicity evaluations have been performed only within limited exposure period of gestation on CPF and were not on Pestban itself. Comprehensive data assessing Pestban effects on length of gestation, reproductive performance and fertility of both males and females has been very limited. Moreover, data addressing its possible adverse effects on lactation exposure are lacking. Therefore, the present study aimed to investigate its effects on the reproductive performance and fertility of both male and female rats, as well as on the incidence of teratogenicity and behavioral effects on the offspring.

## MATERIALS AND METHODS

***Chemicals.*** Pestban (O, O-diethyl-O-3, 5,6-trichloro-2-pyridyl-phosphorothioate), CAS RN/ 2921 – 88 – 2, Developmental code Dowco 179 is one of the commonly used organophosphorus insecticides in Egypt. The commercial formulation used in this study was provided by Agrochem Co. (Alexandria, Egypt). It was available as 48% liquid solution. [$^3$H] acetylcholine iodide (specific activity 55.2 mCi/mmol) was purchased from New England Nuclear (Boston, MA). All other used chemicals were of highest purity and analytical grade.

***Animals.*** Fifty mature males and one hundred mature female Sprague-Dawley rats, 3 months of age and weighing from 180 to 200 g, were used in this study. They were kept under good ventilation and standard hygienic conditions with 12 hour darkness schedule in cages containing four to five animals. Food and water are supplied *ad libitum*.

***Experimental design and administration of the tested chemical.*** The animals were divided into three main groups. The first group consisted of 10 males and twenty females, was given distilled water orally and kept as a control. However, the second and third treated groups were consisted of 20 males and 40 females per group. They were given Pestban by oral intubation at 7.45 and 3.72 mg/kg bwt, equivalent to 1/20 and 1/40 $LD_{50}$, respectively (Worthing and Walker, 1994). Doses were adjusted daily based on weight changes. Male rats were exposed for 70 days; half of the females were dosed for 61 days [14 days premating, during mating and throughout the whole length of gestation, and lactation periods till weaning (21 day after birth)] and the other half exposed only for 40 days (till 20$^{th}$ day of gestation) (Manson and Kang, 1989).

***Examination of male fertility.*** Fertility was estimated in 10 adult exposed males/dose and in another 10 control male counterparts according to the method of Manson and Kang (1989). At the end of the exposure period, each male was placed in a separate cage with two virgin untreated females of the same strain. They were left together for 5 days, during this period, one estrus cycle should be elapsed (Fox and Laird, 1970). The presence of sperms in the vaginal smear was designed as zero day of gestation (Kanojia *et al.*, 1996). The untreated females, which have been paired with treated males, were investigated to evaluate the effects of Pestban exposure on fertility. Half of these untreated females were sacrificed with their fetuses at 20$^{th}$ day of gestation, while the other half was sacrificed with their pups at day 21 of lactation to record the fertility endpoints. The most indicative fertility endpoints according to Manson and Kang (1989) were measured and recorded as following; mating and fertility indices, number of dams showed delayed birth date, signs of dystocia, number of corpora lutea, implantation sites, resorped, dead and live fetuses, pre-and postimplantation losses, dam's body weight at the end of gestation period, gravid uterine and placental weights, fetal body weight (at birth, and at days 4, 7, 14, and 21 after birth), and fetal survival and viability indices during lactation period.

The Pestban exposed males were removed after the mating period and killed by cervical dislocation under light ether anesthesia and the following measurements were recorded: body weight, weights and histopathology of testes, epididymis, prostate and seminal vesicles.

The control male group was mated with 20 untreated females and the endpoints of fertility were recorded in the same manner as aforementioned above in treated male group. In addition, brain tissue samples were taken from the control, treated males, and their offsprings for AChE assay.

***Examination of female fertility.*** Fertility was estimated in 20 adult exposed female rats/ dose and in another 20 control female counterparts according to the method of Manson and Kang (1989). After 14 days of Pestban exposure, each two females were placed in an individual cage with one adult untreated male of proven fertility and of the same strain. They were left together for 5 days, during this period one estrus cycle should be elapsed (Fox and Laird, 1970). The control female counterparts were also caged with untreated males.

The control and Pestban-exposed females were examined for estrus cycle regularity during the premating period (Manson and Kang, 1989). These females were sacrificed (half of them at day 20 of gestation and the other half at day 21 of lactation) with their offspring and the fertility endpoints were recorded in the same manner as previously mentioned in male fertility study. Brain tissue samples of both dams and their offsprings were also taken for AChE assay.

***Determination of acetylcholinesterase activity.*** All frozen (at -70°C) parental (males and females) and fetal brains and placentae were thawed at room temperature and suspended in 50 mM Tris-HCl buffer, pH 7.4 (25°C) containing NaCl, 120 mM; KCl, 5 mM; $CaCl_2$, 2 mM; and $MgCl_2$, 1 mM. Tissues were homogenized (1 : 30, w/v) on ice with a Polytron PT 3000 homogenizer at 28,000 rpm for 20 s. Tissue homogenates were assayed for ChE activity. The enzyme activity was measured by the radiometric method, using a final concentration of 1 mM [3H] acetylcholine iodide (Johnson and Russell, 1975). Each reaction vial (0.1 ml final volume) contained 0.1% Triton X-100 to aid tissue disruption. Preliminary experiments delineated conditions of both incubation time and tissue concentration necessary for linear rates of substrate hydrolysis. Acetylcholinesterase activity was determined by measuring the rate of hydrolysis of acetylcholine iodide ($3 \times 10^{-3}$ M) in buffer (pH 8). Substrate was incubated with brain (2.5 mg wet tissue) in a total volume of 3.2 ml. The absorbance (412 nm) was recorded using a Spectronic 21D Spectrophotometer. Cholinesterase activities were expressed as nmol/min/mg protein. Protein content of all samples was estimated by the method of Lowry *et al*. (1951) using BSA (bovine serum albumin) as a standard.

***Examination of the obtained fetuses.*** All the obtained pups (At 20$^{th}$ day of gestation and at 21$^{st}$ day of lactation) from all groups were examined for presence of any gross malformation and behavioral defects (Manson and Kang, 1989).

One third of the obtained fetuses were preserved in Bouin's solution and examined for presence of any visceral anomalies using the Wilson free hand sectioning technique (Wilson, 1973). The remaining two thirds of the fetuses were preserved in 95% ethanol and examined for presence of any skeletal anomalies according to the method of Manson and Kang (1989).

***Statistical analysis.*** Data presented as percentage were analyzed using Chi-Square test; however, other data were analyzed using one-way ANOVA test. The differences in the data were considered statistically significant at $p < 0.05$ (Norusis, 1994).

## RESULTS

***Clinical signs of toxicity and acetylcholinesterase activity.*** There were no deaths observed during the experimental period. Neither clinical signs nor notable changes in behavior were observed in control or in both treated males and females at the dose of 1/40 $LD_{50}$ (3.72 mg/kg bwt). However, signs of cholinergic toxicity including tremors and salivation were noted in 60% of treated males and in 70% of treated females at the dose rate of 1/20 $LD_{50}$ (7.45 mg/kg bwt). Parental (male and female) and offspring's brain (at 20$^{th}$ day of gestation and after weaning) and placental cholinesterase activities were significantly decreased in a dose related manner with more marked reduction in the treated female groups (Table 1).

***Effects of Pestban on fertility of male and female rats.*** Table 2 demonstrates that the fertility was significantly reduced in untreated female group impregnated with treated males and in treated female group impregnated with untreated males. The estrus cycle regularity was disturbed in the Pestban-exposed females. The mating and fertility indices were significantly reduced in both treated male and female groups compared with

**Table 1.** Effect of Pestban exposure on brain and placental AChE (nmol/mg protein/min) of adult male and female rats and their offsprings

| Groups / Parameters | Control group (10 male + 20 female) | Treated male groups (10 treated male + 20 untreated female/dose) | | Treated female groups (10 untreated male + 20 treated female/dose) | |
|---|---|---|---|---|---|
| | | At 7.45 mg/kg | At 3.72 mg/kg | At 7.45 mg/kg | At 3.72 mg/kg |
| Parental brain AchE[a] | 28.41 ± 6.70 (male & female) | 19.31 ± 4.14[b] (male) | 24.15 ± 6.41 (male) | 16.83 ± 3.98[b] (female) | 21.90 ± 6.57[b] (female) |
| Fetal (At 20th day of gestation) brain AchE[a] | 30.11 ± 8.40 | 27.34 ± 6.86 | 29.45 ± 6.82 | 21.55 ± 6.01[b,c] | 22.14 ± 6.48[b,d] |
| Weaned offsprings brain AchE[a] | 34.66 ± 9.64 | 31.77 ± 9.76 | 32.69 ± 9.80 | 23.56 ± 6.83[b,c] | 30.69 ± 9.13 |
| Placental AchE[a] | 23.13 ± 6.67 | 19.75 ± 4.90 | 21.29 ± 5.85 | 13.10 ± 3.86[b,c] | 16.44 ± 4.55[b,d] |

[a]Values are expressed as mean ± SD.
[b]Significant difference between control and Pestban-treated groups by one-way ANOVA test.
[c]Significant difference between Pestban-treated groups at 7.45 mg/kg bwt by one-way ANOVA test.
[d]Significant difference between Pestban-treated groups at 3.72 mg/kg bwt by one- way ANOVA test.

**Table 2.** Effect of Pestban exposure on fertility and reproductive performance of male and female rats

| Groups / Parameters | | Control group (10 male + 20 female) | Treated male groups (10 treated male + 20 untreated female/dose) | | Treated female groups (10 untreated male + 20 treated female/dose) | |
|---|---|---|---|---|---|---|
| | | | At 7.45 mg/kg | At 3.72 mg/kg | At 7.45 mg /kg | At 3.72 mg/kg |
| No. of females showing regular estrus cycle | | 20 | 20 | 20 | 14 | 16 |
| No. of females with confirmed matings | | 20 | 13 | 15 | 12 | 15 |
| No. of pregnant females | | 18 | 6 | 8 | 10 | 13 |
| Mating index (%) | | 100 | 65[bc] | 75[bd] | 85.71[b] | 93.75 |
| Fertility index (%) | | 95 | 46.15[bc] | 53.33[bd] | 83.33[b] | 86.66[b] |
| No. of dams showed preterm labor (shortened birth date) | | 0 | 4 | 2 | 6 | 4 |
| No. of dams showed signs of dystocia | | 0 | 2 | 1 | 3 | 2 |
| Total No. of corpora lutea | | 203 | 54 | 56 | 74 | 96 |
| Total No. of implantations | | 200 | 38 | 55 | 65 | 92 |
| No. of implants/dam[a] | | 10.51 ± 1.46 | 6.30 ± 1.70[b] | 7.30 ± 1.58[b] | 6.28 ± 1.56[b] | 6.64 ± 1.78[b] |
| No. of resoptions/dam[a] | | 0 | 2.31 ± 0.24[b] | 1.12 ± 0.19[b] | 2.70 ± 0.36[bc] | 1.83 ± 0.56[bd] |
| No. of dead fetuses/dam[a] | | 0.10 ± 0.02 | 1.16 ± 0.19[b] | 1.10 ± 0.17[b] | 1.19 ± 0.31[b] | 1.15 ± 0.26[b] |
| No. of live fetuses/dam[a] | | 10.29 ± 1.61 | 3.92 ± 0.93[b] | 4.92 ± 1.58[b] | 2.38 ± 0.75[bc] | 3.66 ± 0.98[b] |
| Preimplantation loss (%) | | 1.48 ± 0.07 | 29.63 ± 5.14[bc] | 1.78 ± 0.17[b] | 12.16 ± 2.95[b] | 4.16 ± 0.96[bd] |
| Postimplantotion loss (%) | | 4.50 ± 0.80 | 39.47 ± 6.12[b] | 29.09 ± 5.22[b] | 63.07 ± 10.37[bc] | 48.91 ± 8.29[bd] |
| Body weight/dam at termination (g)[a] | | 255.30 ± 24.15 | 225.63 ± 27.94[b] | 226.20 ± 28.06[b] | 220.50 ± 28.79[b] | 223.80 ± 28.47[b] |
| Gravid uterine weight/dam (g)[a] | | 66.40 ± 7.02 | 35.75 ± 8.05[b] | 39.80 ± 9.27[b] | 29.15 ± 3.56[bc] | 32.35 ± 3.95[bd] |
| Placental weight/fetus (g)[a] | | 0.48 ± 0.06 | 0.33 ± 0.16[b] | 0.41 ± 0.15 | 0.26 ± 0.09[b] | 0.28 ± 0.12[bd] |
| Mean | At birth | 5.53 ± 0.47 | 5.31 ± 0.82 | 5.40 ± 0.37 | 4.12 ± 0.63[bc] | 4.42 ± 0.69[bd] |
| | At 4th day pp | 8.02 ± 1.73 | 7.65 ± 1.89 | 7.68 ± 1.90 | 5.10 ± 2.33[bc] | 6.15 ± 2.84[b] |
| Fetal | At 7th day pp | 9.15 ± 2.43 | 8.11 ± 3.03 | 8.70 ± 2.59 | 5.55 ± 2.46[bc] | 6.65 ± 1.76[bd] |
| Weight | At 14th day pp | 12.18 ± 2.84 | 9.41 ± 3.35[b] | 10.26 ± 2.93 | 6.82 ± 3.06[bc] | 9.22 ± 2.84[b] |
| (g)[a] | At 21st day pp | 23.25 ± 4.93 | 18.80 ± 5.37[b] | 20.02 ± 6.16 | 10.14 ± 4.61[bc] | 12.14 ± 6.16[bd] |
| Live/birth index (%) | | 100 | 95.83 | 100 | 91.66 | 97.91 |
| Survival index (%) | | 100 | 95.65 | 100 | 90.91 | 97.87 |
| Viability index (%) | | 99.53 | 91.30 | 97.43 | 81.82 | 95.74 |

PP: Postpartum.
[a]Values are expressed as mean ± SD.
[b]Significant difference between control and Pestban-treated groups by one- way ANOVA test.
[c]Significant difference between Pestban-treated groups at 7.45 mg/kg bwt by one-way ANOVA test.
[d]Significant difference between Pestban-treated groups at 3.72 mg/kg bwt by one-way ANOVA test.

the control group, with more suppression of these indices in the male treated group. These effects occurred in a dose-related manner.

No signs of dystocia or preterm labor (shortened birth date) detected in the control females. However at the highest dose level, 60% of the treated females impreg-

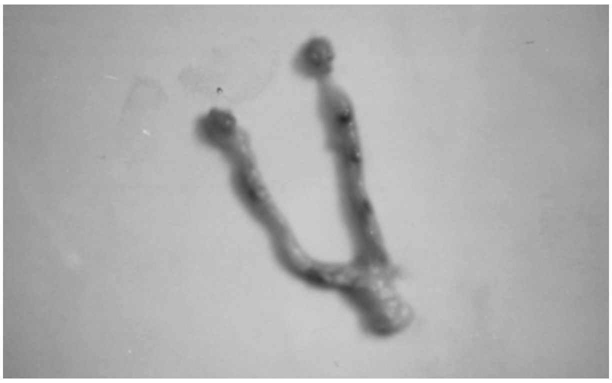

**Fig. 1.** Uterus of pregnant rat exposed to Pestban at 7.45 mg/kg bwt orally and impregnated with untreated males showing postimplantation death.

nated with untreated males showed preterm labor (shortened birth date) and 30% of them showed signs of dystocia compared to 66.66% and 33.33 %, respectively, for the untreated females impregnated with treated males. At the lowest dose, 30.77% of the treated females impregnated with untreated males showed preterm labor and 15.38% of them showed signs of dystocia compared to 25% and 12.5%, respectively, for the untreated females impregnated with treated males.

The number of implantation sites and the number of viable fetuses were significantly reduced in pregnant females of both treated groups at the two dose levels compared with the control group. However, the number of resorptions (postimplantation deaths) (Fig. 1), dead fetuses and pre-and postimplantation losses were significantly increased in pregnant females of both treated groups. The incidence of resorptions was significantly increased with increasing the dose in treated female group impregnated with untreated males in comparison with untreated female group impregnated with treated males.

The placental, fetal, and gravid uterine weights were significantly decreased in both treated groups at the two dose levels compared with control group. The body weight of fetuses obtained from treated females impregnated with untreated males was significantly different (at birth and at the end of lactation period) from those obtained from untreated females impregnated with treated males.

During lactation, the pups behavioral responses (such as memory response), fetal survival and viability indices were decreased in both treated groups. The incidence of these effects was more pronounced in the treated female group impregnated with untreated males compared with untreated female group impregnated with treated males. At the highest dose, 50% of the offsprings obtained from the treated female group impregnated with untreated males showed mild cholinergic toxicity signs during lactation period such as tremors and salivation. These signs were not observed in the offsprings obtained from the untreated females impregnated with treated males. However at the lowest dose, no cholinergic toxicity signs appeared on the offsprings obtained from all treated groups.

***Effects of Pestban on sex organ weights and histological structure of treated males.*** The body weight of male rats exposed to Pestban either at 7.45 or 3.72 mg/kg showed no significant alteration. However, the weights of testes, epididymis, prostate, and seminal vesicles were significantly reduced in a dose-dependant manner compared with the control ones (Table 3).

**Table 3.** Body and sex organs weight (g) of adult male rats exposed to Pestban for 70 successive days

| Groups / Parameters | Control males | Treated males At 7.45 mg/kg | Treated males At 3.72 mg/kg |
|---|---|---|---|
| Number of males | 10 | 10 | 10 |
| Body weight[a] | 215 ± 5.21 | 197 ± 5.05 | 198 ± 5.37 |
| Testes weight[a] | 3.85 ± 0.56 | 2.70 ± 1.04[b] | 3.05 ± 0.79[b] |
| Epididymis weight[a] | 0.64 ± 0.15 | 0.43 ± 1.04[b] | 0.54 ± 0.22 |
| Prostate gland weight[a] | 0.55 ± 0.06 | 0.41 ± 0.09[bc] | 0.49 ± 0.12 |
| Seminal vesicles weight[a] | 1.20 ± 0.27 | 0.87 ± 0.22[b] | 0.98 ± 0.25 |

[a]Values are expressed as mean ± SD.
[b]Significant difference between control and Pestban-treated groups by one-way ANOVA test.
[c]Significant difference between the two Pestban-treated groups by one-way ANOVA test.

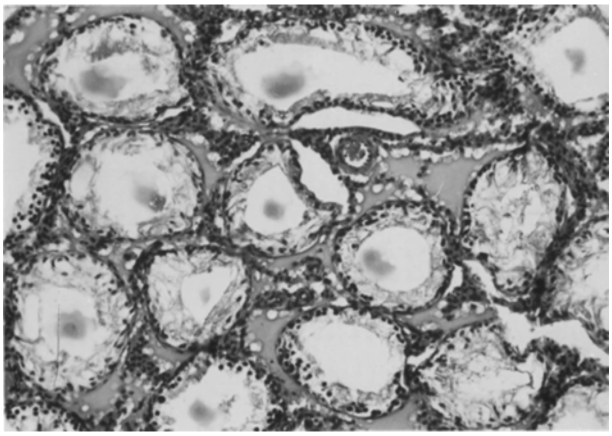

**Fig. 2.** Photomicrograph of the testis of rat exposed orally to 7.45 mg/Kg bwt Pestban daily for 60 days showing atrophied and degenerated seminiferous tubules with edema and hyperplasia of the interstitial Leydig cells (H& E, × 40).

Histopathological examination revealed different alterations in the sex organs of Pestiban-exposed animals including the testes, epididymis, prostate gland, and seminal vesicles. The histopathological lesions occurred in a dose-dependant manner. The seminiferous tubules were atrophied and showed degenerative changes in the germinal epithelium, desquamation of spermatogenic cells with appearance of Sertoli cells as predominating cells (Fig. 2) and hyperplasia of the interstitial Leydig cells (Fig. 3), at the high dose-exposed animals after 70 days exposure compared to control rat's testes. Moreover, vacuolation and degeneration of the seminiferous tubules (Fig. 4) were also seen at the low dose-exposed animals.

The epithelial lining of epididymal ducts showed mild hyperplasia with moderate inflammatory cells infiltration and edema (Fig. 5) in the high dose-exposed animals. However, it showed only mild inflammatory cells infiltration in the low dose-treated group.

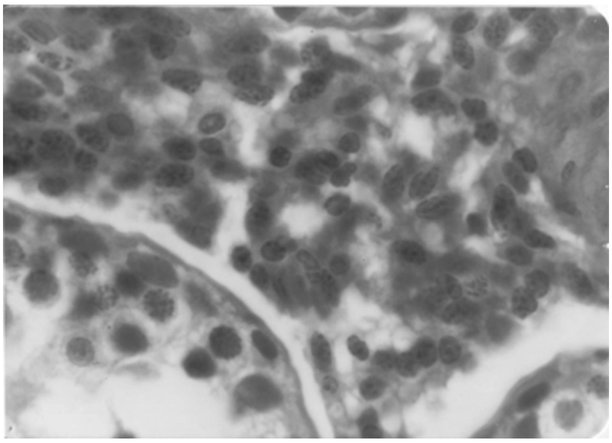

**Fig. 3.** A cross section in the testis of rat exposed orally to 7.45 mg/kg bwt Pestban daily for 60 days showing hypertrophy and hyperplasia of the interstitial Leydig cells (H& E, × 1000).

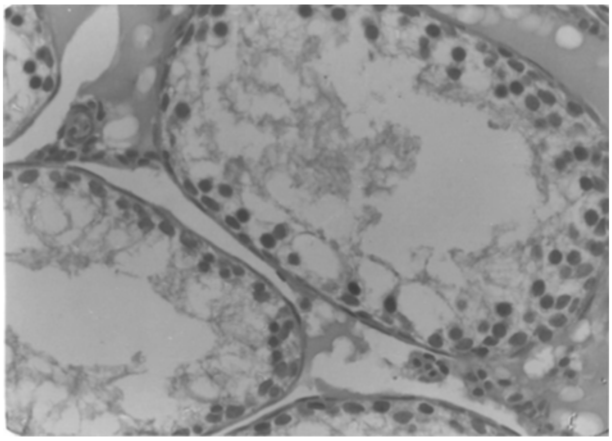

**Fig. 4.** A cross section in the testis of rat exposed orally to 3.72 mg/kg bwt Pestban daily for 60 days showing vacuolated and degenerated seminiferous tubules (H& E, × 160).

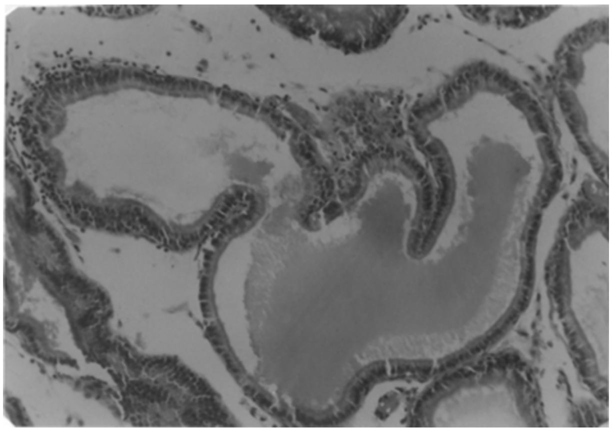

**Fig. 5.** A section in the epididymis of rat exposed orally to 7.45 mg/kg bwt Pestban daily for 60 days showing mild hyperplasia of the lining epithelium with moderate inflammatory cells infiltration and edema (H& E, × 160).

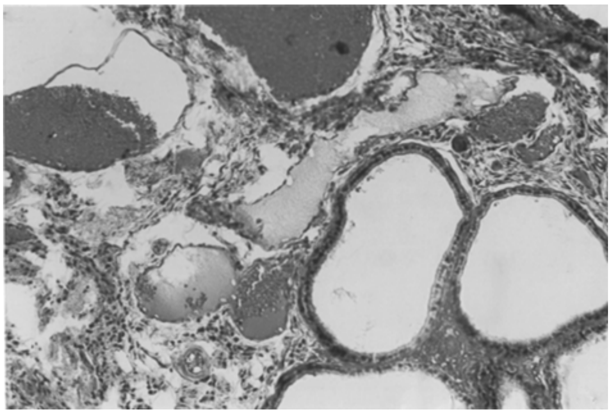

**Fig. 6.** A section in the prostate gland of rat exposed orally to 7.45 mg/kg bwt Pestban daily for 60 days showing massive numbers of dilated blood capillaries in the stromal tissue (Hemangioma) (H& E, × 40).

The prostate gland of the high dose-treated animals showed massive numbers of dilated blood capillaries in the stromal tissue (Hemangioma) (Fig. 6). On the other hand, it showed mononuclear inflammatory cells infiltration, edema, and dilated blood capillaries in the interacinar stroma with hyperplasia of its epithelial lining (Fig. 7) at the low dose of exposure. The seminal vesicle showed only mild inflammatory cells infiltration at the high dose-exposed animals.

***Effects of Pestban on the incidence of developmental anomalies in the obtained fetuses.*** Gross examination of the fetuses of both treated groups

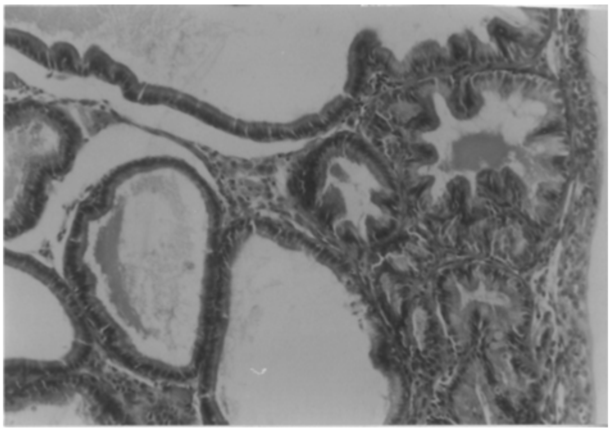

**Fig. 7.** Photomicrograph of the prostate gland of rat exposed orally to 3.72 mg/kg bwt Pestban daily for 60 days showing mononuclear inflammatory cells infiltration with oedema and dilated blood capillaries in the interacinar stroma and hyperplasia of the epithelial lining of the prostatic acini (H& E, × 160).

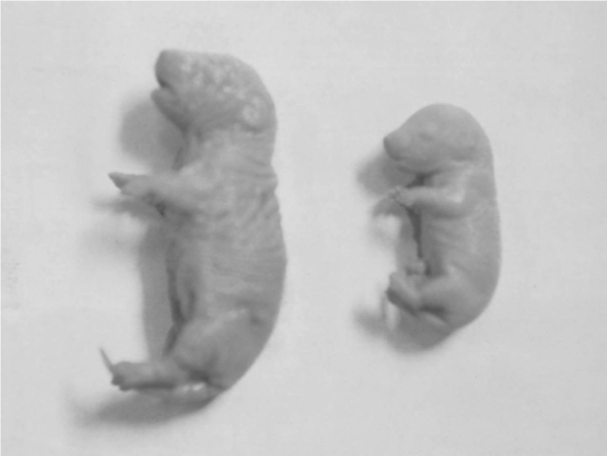

**Fig. 8.** A rat fetus obtained from an untreated dam impregnated with males exposed to Pestban at 3.72 mg/kg bwt orally for 60 days daily showing stunted growth (right) and a control fetus (left).

**Table 4.** Incidences of gross anomalies in the fetuses obtained from Pestban-exposed male and female rat groups

| Groups | Fetuses of control group | Fetuses of treated male group | | Fetuses of treated female groups | |
|---|---|---|---|---|---|
| Parameters | | At 7.45 mg/kg | At 3.72 mg/kg | At 7.45 mg/kg | At 3.72 mg/kg |
| Total fetuses examined | 191 | 23 | 39 | 22 | 47 |
| Stunted growth[a] | 0 | 21.74[b] | 10.26[b] | 59.09[bc] | 29.79[bd] |
| Exencephaly[a] | 0 | 17.23[b] | 7.69[b] | 54.54[bc] | 27.66[bd] |
| Generalized edema[a] | 0 | 13.04[b] | 5.13[b] | 45.45[bc] | 25.53[bd] |
| Micrognathia[a] | 0 | 8.69[b] | 5.13[b] | 40.90[bc] | 21.28[bd] |

[a]Values represent percentage of grossly deformed fetuses in relation to total number of fetuses examined.
[b]Significant difference between control and Pestban-treated groups by Chi-Square test.
[c]Significant difference between Pestban-treated groups at 7.45 mg/kg bwt by Chi-Square test.
[d]Significant difference between Pestban-treated groups at 3.72 mg/kg bwt by Chi-Square test.

**Table 5.** Incidences of visceral anomalies in the fetuses obtained from Pestban-exposed male and female rat groups

| Groups | Fetuses of control group | Fetuses of treated male group | | Fetuses of treated female groups | |
|---|---|---|---|---|---|
| Parameters | | At 7.45 mg/kg | At 3.72 mg/kg | At 7.45 mg/kg | At 3.72 mg/kg |
| Total fetuses examined | 64 | 8 | 13 | 8 | 16 |
| Total fetuses with visceral anomalies | 2 | 3 | 2 | 7 | 9 |
| Total fetuses with head anomalies | 0 | 2 | 1 | 5 | 5 |
| -Dilated brain ventricles[a] | 0 | 12.25[b] | 7.69[b] | 62.50[bc] | 18.75[bd] |
| -Dilated nares[a] | 0 | 25.00[b] | 7.69[b] | 50.00[bc] | 25.00[bd] |
| -Olfactory pulp hypoplasia[a] | 0 | 0 | 0 | 25.00[bc] | 18.75[bd] |
| -Cerebral hemisphere hypoplasia[a] | 0 | 25.00[b] | 7.69[a] | 62.50[bc] | 25.00[bd] |
| -Micro-and anopthalmia[a] | 0 | 12.25[b] | 0 | 50.00[bc] | 31.25[bd] |
| Total fetuses with thorax anomalies | 1 | 3 | 3 | 7 | 8 |
| -Heart hypertrophy[a] | 0 | 37.50[b] | 23.07[b] | 50.00[bc] | 37.50[bd] |
| -Lung hypoplasia[a] | 0 | 25.00[b] | 15.38[b] | 87.50[bc] | 50.00[bd] |
| -Intrathoracic hemorrhages[a] | 1.56 | 12.25[b] | 7.69[b] | 62.50[bc] | 43.75[bd] |
| Total fetuses with pelvic anomalies | 2 | 3 | 2 | 6 | 7 |
| -Dilated renal pelvis[a] | 3.12 | 37.50[b] | 15.38[b] | 75.00[bc] | 43.75[bd] |
| -Hydroureter[a] | 0 | 12.25[b] | 7.69[b] | 25.00[bc] | 12.50[bd] |

[a]Values represent percentage of viscerally deformed fetuses in relation to total number of fetuses examined.
[b]Significant difference between control and Pestban-treated groups by Chi-Square test.
[c]Significant difference between Pestban-treated groups at 7.45 mg/kg bwt by Chi-Square test.
[d]Significant difference between Pestban-treated groups at 3.72 mg/kg bwt by Chi-Square test.

obtained at 20$^{th}$ day of gestation and after weaning revealed significant increase in the percentage of stunted growth (Fig. 8), exencephaly, generalized edema, and micrognathia. The incidence of these fetal anomalies was more pronounced in the fetuses of treated female group than in the fetuses of treated male group at the two dose levels (Table 4).

Pestban significantly increased the incidence of visceral anomalies in the fetuses of both treated groups compared with control fetuses (Table 5). These anomalies were in the form of dilated brain ventricles (hydrocephaly) (Fig. 9), dilated nares, olfactory bulb and cerebral hemisphere hypoplasia, micro-and anophthalmia (Fig. 10), intrathoracic hemorrhage, heart and lung hypoplasia (Fig. 11), hydroureter, renal hypoplasia, and dilated renal pelvis (Fig. 12). The incidence of these visceral anomalies was greater in the fetuses of treated female group compared with fetuses of treated male group especially at the high dose of this insecticide.

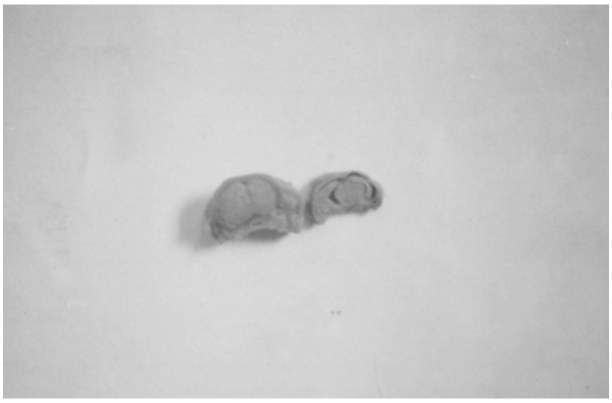

**Fig. 9.** A transverse section in the head of a rat fetus obtained from a dam exposed to Pestban at 7.45 mg/kg bwt orally and impregnated with untreated males showing dilated brain lateral ventricles (right) and a control one (left).

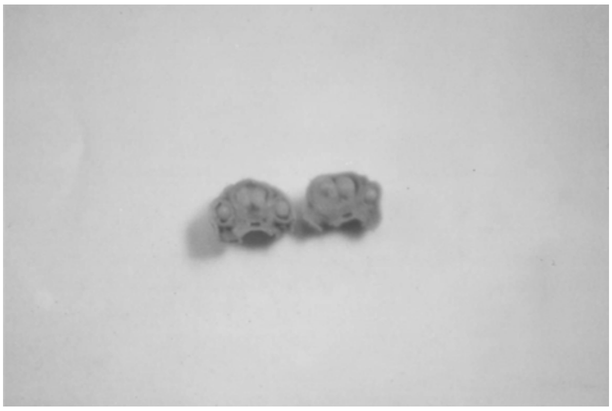

**Fig. 10.** A transverse section in the head of a rat fetus obtained from an untreated dam impregnated with males exposed to Pestban at 3.72 mg/kg bwt orally for 60 days daily showing anophthalmia (right) and a control one (left).

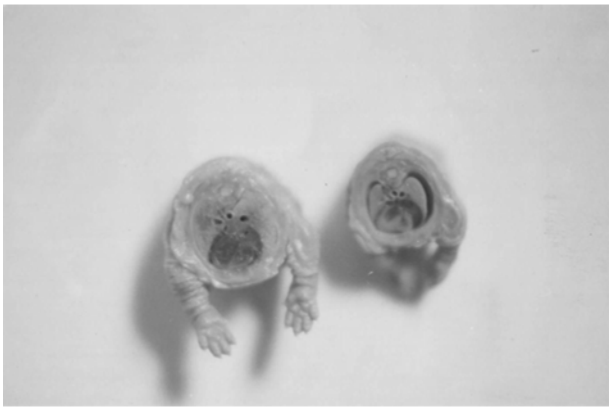

**Fig. 11.** A transverse section in the chest of a rat fetus obtained from a dam exposed to Pestban at 7.45 mg/kg bwt orally and impregnated with untreated males showing intrathoracic hemorrhages; lung and heart hypoplasia (right) and a control one (left).

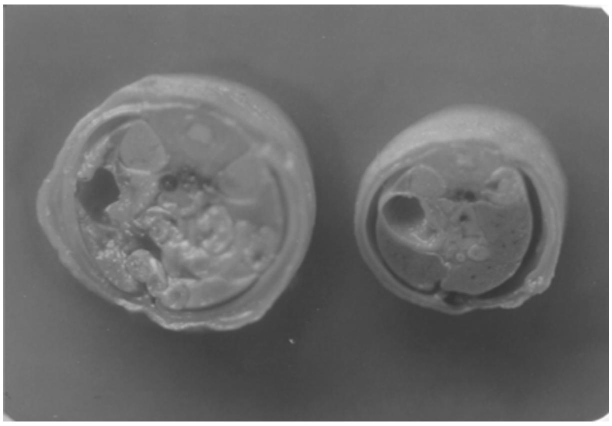

**Fig. 12.** A transverse section near the pelvic region in a weaned rat fetus obtained from an untreated dam impregnated with males exposed to Pestban at 7.45 mg/kg bwt orally for 60 days daily showing dilated renal pelvis (right) and a control one (left).

The incidence of skeletal anomalies in the fetuses of both treated groups was significantly increased compared with the control fetuses (Table 6). The recorded skeletal anomalies were in the form of wide separation of parietal bones, incomplete ossification of parietal and /or interparietal bones (Fig. 13), incomplete ossification of sternebrae, reduced sternbrae number (Fig. 14), wavy and extra ribs, absence of carpal and metacarpal, tarsal and metatarsal bones, absence of caudal bones and phalanges (Fig. 13). The incidence of these skeletal anomalies was more pronounced in fetuses of treated female group than in the fetuses of treated male

**Table 6.** Incidences of skeletal anomalies in the fetuses obtained from Pestban-exposed male and female rat groups

| Groups / Parameters | Fetuses of control group | Fetuses of treated male group At 7.45 mg/kg | Fetuses of treated male group At 3.72 mg/kg | Fetuses of treated female groups At 7.45 mg/kg | Fetuses of treated female groups At 3.72 mg/kg |
|---|---|---|---|---|---|
| Total fetuses examined | 127 | 15 | 26 | 14 | 31 |
| Total fetuses with skeletal anomalies | 2 | 6 | 4 | 13 | 18 |
| Total fetuses with skull anomalies | 0 | 4 | 2 | 11 | 8 |
| - Wide separation of parietal bones[a] | 0 | 13.33[b] | 0 | 50.00[b,c] | 19.35[b,d] |
| - Incomplete ossification of parietal bones[a] | 0 | 20.00[b] | 3.85[a] | 78.75[b,c] | 25.81[b,d] |
| - Incomplete ossification of interparietal bones[a] | 0 | 26.66[b] | 7.69[a] | 64.28[b,c] | 22.58[b,d] |
| Total fetuses with sternebrae anomalies | 1 | 5 | 3 | 10 | 10 |
| - Incomplete ossification of sternebrae[a] | 0 | 13.33[b] | 11.54[a] | 64.28[bc] | 22.58[b,d] |
| - Reduced sternebrae number[a] | 0.79 | 33.33[b] | 11.54[a] | 71.43[b,c] | 32.26[b,d] |
| - Deformed ribs[a] | 0 | 26.66[b] | 7.69[a] | 50.00[b,c] | 25.81[b,d] |
| - Extra ribs[a] | 0 | 26.00[b] | 3.85[a] | 35.71[b,c] | 19.35[b,d] |
| Total fetuses with limb anomalies | 2 | 6 | 4 | 12 | 14 |
| - Absence of carpal and/or metacarpal bones[a] | 0 | 40.00[b] | 15.38[a] | 78.75[b,c] | 41.94[b,d] |
| - Absence of tarsal and/or metatarsal bones[a] | 0 | 33.33[b] | 11.54[a] | 71.43[b,c] | 38.71[b,d] |
| - Absence of phalanges[a] | 1.57 | 20.00[b] | 7.69[a] | 85.71[b,c] | 45.61[b,d] |
| - Absence of caudal bones[a] | 0 | 26.66[b] | 7.69[a] | 50.00[b,c] | 29.03[b,d] |

[a]Values represent percentage of skeletally deformed fetuses in relation to total number of fetuses examined.
[b]Significant difference between control and Pestban-treated groups by Chi-Square test.
[c]Significant difference between Pestban-treated groups at 7.45 mg/kg bwt by Chi-Square test.
[d]Significant difference between Pestban-treated groups at 3.72 mg/kg bwt by Chi-Square test.

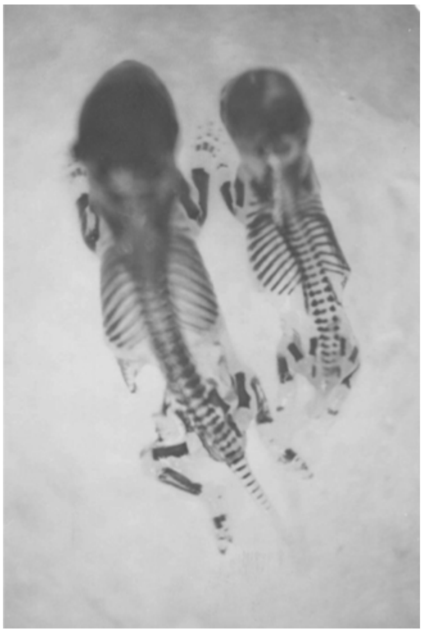

**Fig. 13.** A rat fetus obtained from an untreated dam impregnated with males exposed to Pestban at 7.45 mg/kg bwt orally for 60 days daily showing incomplete ossification of skull bones and phalanges as well as absence of sacral and coccygeal vertebrae.

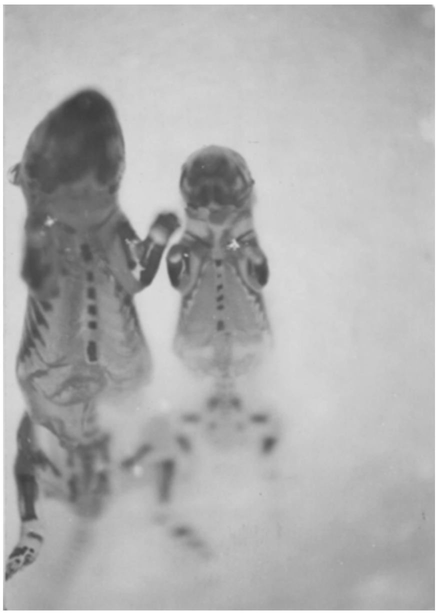

**Fig. 14.** A rat fetus obtained from a dam exposed to Pestban at 3.72 mg/kg bwt orally and impregnated with untreated males showing incomplete ossification of sternbrae (right) and a control fetus (left).

group and occurred in a dose-related manner.

## DISCUSSION

The aim of the present study was to monitor the adverse effects of Pestban on fertility and reproduction of both male and female rats. Generally there was no mortality or clinical signs of toxicity in any of the exposed male or female groups at the lowest dose (3.72 mg/kg). However, signs of cholinergic toxicity including tremors and salivation were noted in 60% of treated males and in 70% of treated females at the highest dose (7.45 mg/kg). This was associated by the significantly inhibited brain AChE activity in the highest Pestban-treated groups compared to control and lowest Pestban-treated groups.

The obtained results showed that exposure of male and female rats to Pestban had adverse effects on their reproductive performance and fertility. Similarly, exposure of rabbits to CPF orally at 0.1 and 0.05 mg/kg daily for 70 and 30 days, respectively reduced male and female fertility with severe ovarian and testicular histopathological alterations (Bansal *et al*., 1994). The observed inhibition of parental and neonatal AChE activities, reproductive and neonatal adverse effects following Pestban exposure agree with the results of Astroff *et al*. (1998) and Farag *et al*. (2003, 2006) in rats following exposure to either tribufos, oxydemeton-methyl, fenamiphos, coumaphos, trichlorfon, CPF or dimethoate as OPI. Pestban-induced AChE suppression may be due to adverse effects on neuronal activity which normally regulates critical genes such as the neurotrophins nerve growth factor (NGF) and brain-derived neurotrophic factor (BDNF) (Betancourt *et al*., 2007).

Testicular lesions due to organophosphates exposure were also recorded by Carleton *et al.* (1987); Somkuti *et al.* (1991) and Piramanayagam *et al.* (1996) in rats, Wenda-Rozewicka (1984) and Chapin *et al*. (1988) in mice, and Bansal *et al.* (1994) in rabbits. In addition, the recorded Pestban-induced adverse effects on testis, accessory sex glands, male reproductive performance, and fertility are in coincidence with the results of Ray *et al.* (1991, 1992); Cho and Park (1994) and Piramanayagam *et al*. (1996) in exposed adult rats to quinalphos; dimethyl methylphosphonate (DMMP) or trimethylphosphate (TMP), and malathion, respectively. Moreover, Debnath and Mandal (2000); Kamijima *et al*. (2004) and Okamura *et al*. (2005) confirmed the male reproductive toxicity of OPI in animals and humans with the induction of testicular lesions. Narayana *et al.* (2006) mentioned that neonatal {on postnatal day (PND) 3 to PND 28} oral methyl parathion exposure of rat affected the growth and functions of the male reproductive system in the adult life.

AChE and butyrylcholinesterase (BuChE) activities have been identified in mouse and rat Sertoli cells and spermatozoa (Chakraborty and Nelson, 1976). The observed antifertility effect of Pestban could be attributed to its AChE inhibiting activity and the consequent suppression of sperm motility which is dependent on presence of acetylcholine (Harbison *et al*., 1976; Abou-Donia, 1985). Studies have shown that antiandrogenic activity (Tomura *et al*., 2001; Kitamura *et al*., 2003; Kojima *et al*., 2004); altered androgen metabolism (Hodgson and Rose, 2006); pathological changes in the testes (Chapin *et al*., 1988); mutagenic potential on spermatogonial cells (El Nahas *et al*., 1989) and suppressive effect on the functional activity of accessory sex glands (Ray *et al*., 1991) may be responsible for decreased reproductive performance in animals exposed to organophosphates. Moreover, Sanchez-Pena *et al.* (2004) suggested that OPI exposure alters sperm chromatin condensation, which could be reflected in an increased number of cells with greater susceptibility to DNA denaturation and may contribute to adverse reproductive outcomes. Therefore, the declined mating and fertility indices of the treated males following Pestban exposure may be due to its adverse effect on AChE; spermatogonial DNA, androgen metabolism; accessory sex glands and/or testicular function. This was confirmed by the recorded adverse effects on the weight and histological structures of the male sex organs.

Administration of 3.72 mg/kg per day Pestban to adult female rats by gavage did not produce maternal toxicity but induced embryo-/feto-toxicity and teratogenicity. These results are consistent with the previous studies that addressed the potential embryotoxicity and teratogenicity of anticholinesterase compounds (Lozano *et al*., 1989; Soni and Bhatngar, 1989; Weber, 1990; Srivastava *et al*., 1992; Chanda *et al*., 1995; Srivastava and Raizada, 1996; Dam *et al*., 1998; Farag *et al*., 2003; Akhtar *et al*., 2006). Developmental toxicity was observed in the two treated groups at the two dose levels with suppressed placental, maternal, and fetal brain cholinesterase activity especially in the treated female group at the high dose level. The recorded AChE suppressive properties of Pestban probably contributed to its embryolethality and developmental toxicity (Lassiter *et al*., 1998; Slotkin, 1999). In addition, the inhibition of fetal brain cholinesterase may be deleterious to the coordinated development of the brain given the postulated novel role for the cholinesterases in nervous system development (Lassiter *et al*., 1998). Chlorpyrifos disrupted the maturation of sea urchin embryos during the specific period during which development is regulated by neurotrophic factors (Buznikov *et al*., 2001). Studies suggested that CPF may directly influence brain cell replication and differentiation (Dam *et al*., 1998; Crumpton *et al*., 2000). Chlorpyrifos has immediate direct inhibitory actions on DNA synthesis and hence on neural cell replication, with preferential targeting of gliotypic cells (Lassiter *et al*., 1998). Therefore, chlorpyrifos may induce damage by both noncholinergic and cholinergic mechanisms extending from early stages of neural cell replication through late stages of axonogenesis and terminal differentiation (Slotkin, 1999).

The number of implantation sites and the number of viable fetuses were significantly reduced in pregnant females of both treated groups at the two dose levels. However, the number of resorptions, dead fetuses and pre-and postimplantation losses were significantly increased. Organophosphorus compounds have been reported to induce early and late embryonic deaths (Chanda *et al.*, 1995; Srivastava and Raizada, 1996; Dam *et al.*, 1998; Farag *et al.*, 2003; Mahadevaswami and Kaliwal, 2003). The Pestban-induced implantation delay and nidation could be attributed to an imbalance in the estrogen-progesterone ratio, which is essential for implantation (Mahadevaswami and Kaliwal, 2003). Alternatively, Pestban treatment could result in blastotoxicity and/or have an impact on the hypothalamic-pituitary axis.

The incidence of resorptions was significantly increased in treated female group impregnated with untreated males compared with untreated female group impregnated with treated males. The observed increase in embryonal resorptions in female rats exposed to Pestban most probably resulted from modification of the uterine lining function and/or transplacental Pestban passage with subsequent AChE inhibition (Srivastava *et al.*, 1992; Slotkin, 1999; Farag *et al.*, 2003; Akhtar *et al.*, 2006).

During the lactation period, the fetal body weight, survival and viability indices, and the behavioral responses such as memory response and brain AChE were significantly decreased in the treated female group impregnated with untreated males compared with untreated female group impregnated with treated males. It has been reported that lactation exposure of suckling mice to malathion through maternal milk caused a high inhibitory effect of the brain AChE activity in the offspring summing to the fact that OPI are excreted in milk (Silva *et al.*, 2006).

Similar effects to those of Pestban on the pup viability and survival were observed by Carleton *et al.* (1987) after daily oral treatment of both male (for 56 days) and female (for 14 days prior to mating and throughout the mating period, gestation and lactation) rats with tricresyl phosphate (TCP). Furthermore, it has been reported that malathion exposure reduced the size of rat litters and decreased the survival rate of the young (Harte *et al.*, 1991). The fetal growth retardation and neonatal deaths (decreased survival and viability indices) in rats exposed to organophosphates could be a consequence of alteration of the milk lipase activity with a diminished secretory function in the mammary gland leading to interference with nursing of the offsprings (Fish, 1966). Moreover, Pestban is a potent inhibitor of DNA and protein synthesis that could affect several metabolic processes (Nehez and Desi, 1996; Blasiak *et al.*, 1999; Eskenazi *et al.*, 1999). These effects might be responsible for Pestban-induced fetal growth retardation and developmental toxicity.

The observed behavioral disturbances were similar to the results of Eskenazi *et al.* (1999). Richardson and Chambers (2004, 2005) found that gestational and postnatal oral exposure of rat to 1.5-7 mg/kg/day CPF resulted in long-term alterations of presynaptic cholinergic neurochemistry in developing pups. These behavioral disturbances could be attributed to the effects of Pestban on the central nervous system, most importantly, alteration in the content of AChE, which participate in the morphological process and the maturation of central nervous system (Lassiter *et al.*, 1998; Slotkin, 1999), down-regulation of muscarinic receptors and decreased brain DNA synthesis (Eskenazi *et al.*, 1999). Additionally, it might be due to the generation of free radical in the developing rat brain (Gupta *et al.*, 1998).

The incidences of the recorded gross, visceral, and skeletal anomalies were significantly increased in the fetuses obtained from treated female group compared with the fetuses of treated male group with significantly increased percentages at the high dose level. This could be attributed to transplacental transfer of Pestban to the fetuses from exposed pregnant females during the organogenesis period (Srivastava *et al.*, 1992; Farag *et al.*, 2003; Akhtar *et al.*, 2006). The organogenesis period considered the most sensitive period of fetal developmental abnormalities (Leone, 1977). The placenta may be directly involved in many instances of early spontaneous abortions, fetal death, and intrauterine growth retardation (Faulk, 1981). The associations between ChE levels in placenta and parameters of fetal growth and length of gestation are in agreement with the results of Eskenazi *et al.* (2004). Lower levels of ChE in placenta were associated with significantly shorter length of gestation. Decreasing the levels of placental ChE were also associated with increased incidences of dystocia, preterm labour (shortened birth date) and low birth weight. Moreover, placental ChE levels were also associated with most of the other fetal parameters and birth outcome. Cholinergic nerves play a significant role in the control of the uterine musculature and myometrium. Acetylcholine stimulates contraction of the uterus and dilates its arterial supply (Papka *et al.*, 1999). Thus, an inhibition of acetyl ChE could produce an accumulation of acetylcholine in the neuronal junctions and hence the over-stimulation of cholinergic fibers resulting in premature initiation of labour. In addition, premature delivery may be the cause of decreased survival and viability of the fetuses during lactation.

Many trends in teratogenicity research have focused on the possibility that the disruption in morphogenesis may be related to alkylation of nucleic acids by organophosphates (Bedford and Robinson, 1972). It has been postulated that alkylation of NAD coenzymes by organophosphates may be a major factor in the induction of teratogenesis (Schoental, 1977). Other investigators have found altered levels of RNA, glycogen, sulfated mucopolysaccharides and calcium by organophosphates in the developing tibiotarsus of chick embryo (Ho and Gibson, 1972).

In conclusion, the present study indicates that oral exposure to Pestban by male and female rats during the different phases of reproduction affect male and female fertility with teratogenic and postnatal effects on the growing fetuses. The fetal developmental effects were greater in treated females compared with untreated females impregnated with treated males and occurred in a dose related manner.

## ACKNOWLEDGMENTS

Grateful thanks offered to Dr. A. Bakeer, Prof. of Pathology, Faculty of Veterinary Medicine, Cairo University for his help in the histopathological examination.
